# Complete Genome Sequence of *Caballeronia* sp. Strain NK8 (MAFF311271), a Chlorobenzoate-Degrading Bacterium

**DOI:** 10.1128/MRA.00416-21

**Published:** 2021-08-05

**Authors:** Kimiko Yamamoto-Tamura, Ryota Moriuchi, Naoto Ogawa

**Affiliations:** a Institute for Agro-Environmental Sciences, National Agriculture and Food Research Organization, Tsukuba, Japan; b Research Institute of Green Science and Technology, Shizuoka University, Shizuoka, Japan; c Graduate School of Integrated Science and Technology, Shizuoka University, Shizuoka, Japan; Montana State University

## Abstract

*Caballeronia* sp. strain NK8 grows on 3-chlorobenzoate and shows chemotaxis toward 3-chlorobenzoate and its degradation products, such as chlorocatechols. Complete genome sequencing revealed a 9.2-Mb genome consisting of three chromosomes and four plasmids. The genes for degradation of 3-chlorobenzoate and chlorocatechols were located on plasmids pNK81 and pNK84, respectively.

## ANNOUNCEMENT

*Caballeronia* sp. strain NK8 (formerly *Burkholderia* sp. strain NK8) (= MAFF311271) was isolated from a soil sample in Japan (1). NK8 can degrade 3-chlorobenzoate (3CB), an intermediate of polychlorinated biphenyls, via the chlorocatechol *ortho*-cleavage pathway encoded by the *tfdCDEF* genes (2). We previously demonstrated that NK8 cells are motile and exhibit chemotaxis toward 3CB and its degradation products (3).

A single colony of NK8 (our laboratory stock strain) grown on an agar plate of basal salts medium (4) containing 5 mM 3CB was inoculated into the same medium without agar and grown at 28°C for 3 days. The cells were harvested, and the genomic DNA was prepared using a Wizard genomic DNA purification kit (Promega) and a Qiagen blood and cell culture DNA maxikit (Qiagen) for subsequent sequencing with an Illumina HiSeq 2000 system (Illumina) and an RS II system (Pacific Biosciences [PacBio]), respectively. Genome sequencing was performed at the Beijing Genome Institute (BGI). HiSeq libraries of 500-bp size were prepared using the BGI in-house method described below. Purified genomic DNA was fragmented randomly with a Covaris S/E210 or Bioruptor sonicator, and then the DNA fragments of the required length were retained by electrophoresis. After this, adapters were ligated to the DNA fragments, and the fragments were selectively enriched and amplified by PCR. The PacBio library was constructed using a SMRTbell template preparation kit v. 1.0 according to the PacBio 20-kb library protocol. PacBio subreads were filtered (read quality of >0.85) using BamTools v. 2.5.1 (5), and the resulting subreads, which consisted of 91,368 reads with a maximum read length of 39,991 bp and an *N*_50_ value of 11,303 bp (total of 745,616,458 bp, with 81-fold genome coverage), were used for *de novo* assembly in Canu v. 1.8 (6) with the genomeSize = 9.2m setting. The resulting contigs were polished using Arrow (https://github.com/PacificBiosciences/GenomicConsensus), and then overlapping regions at both ends of each contig were confirmed and trimmed with Circlator v. 1.1.1 (7). The seven closed contigs obtained were polished using Pilon v. 1.23 (8) with trimmed Illumina paired-end short reads (14,313,650 reads totaling 1,431,365,000 bp, with 155-fold genome coverage) that had been obtained from 2 × 101-bp paired-end read sequencing. Illumina reads were cleaned by Trimmomatic v. 0.39 (9) (read length, ≥100 bp; quality score, ≥15). Genome annotation was performed using DFAST v. 1.2.7 (10). Default parameters were used for all software unless otherwise specified.

**FIG 1:**
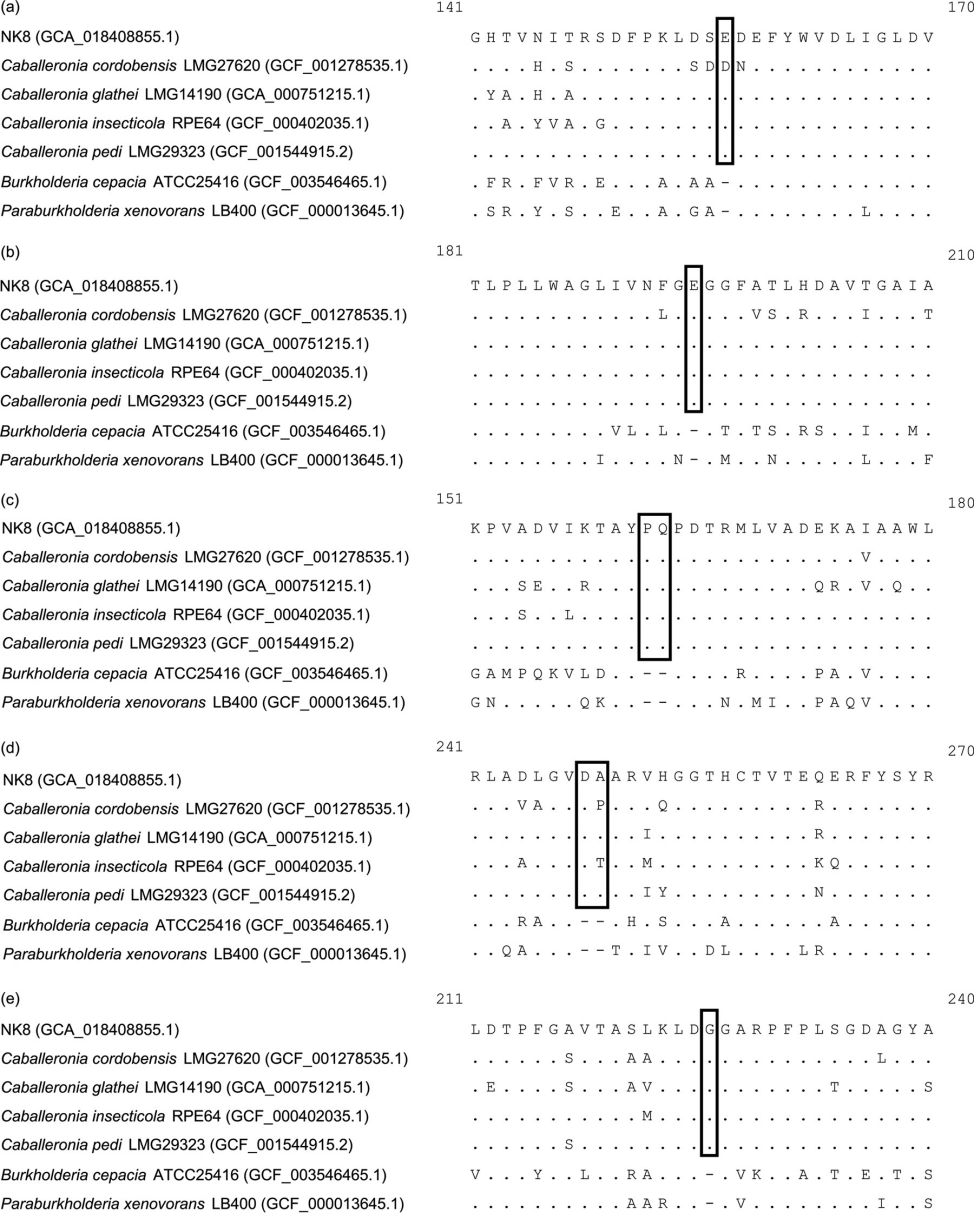
Putative amino acid sequence alignments of 16S rRNA-processing protein RimM (a), prepilin peptidase (b), cytochrome oxidase subunit I (c), peptidoglycan editing factor PgeF (d), and translocation and assembly module protein TamB (e), showing *Caballeronia-*specific CSIs (boxed). Except for NK8, strains used for this analysis were type strains of the species. The NCBI assembly accession number for each strain is shown in parentheses.

The NK8 genome consisted of three chromosomes and four plasmids (Table 1). The genes for the conversion of 3CB to 3- and 4-chlorocatechols (*cbeABCD*) were located on pNK81. The *tfdCDEF* genes (NK8_82450 to NK8_82480) (2) were on pNK84 along with the putative DNA relaxase gene *traI* (NK8_85450) (3), suggesting that the *tfdCDEF* genes were acquired by horizontal transfer. The genus *Caballeronia* has recently been divided from the genus *Burkholderia* (11). Conserved sequence indels (CSIs) can be used as *Caballeronia-*specific molecular markers to distinguish the genus from other related bacteria (12). Using a similar approach, we revealed specific CSIs in the putative amino acid sequences of NK8 (Fig. 1). The analysis of two-way average nucleotide identity (ANI) using an ANI calculator (http://enve-omics.ce.gatech.edu/ani) revealed that Caballeronia pedi LMG 29323 (NCBI BioProject accession number PRJEB12492) was the closest to NK8 among the type strains of *Caballeronia*, with an identity of 94.85%, which was slightly lower than the ANI cutoff value generally accepted for species delineation (95 to 96%) (13, 14). Furthermore, while NK8 showed chemotaxis (3), LMG 29323 was described as nonmotile by Peeters et al. (15). These findings suggest that NK8 represents a new species of the genus *Caballeronia*.

**TABLE 1:** Summary of the assembly and annotation statistics of *Caballeronia* sp. strain NK8

Genetic element	Size (bp)	GC content (%)	No. of coding sequences	No. of rRNAs	No. of tRNAs	DDBJ accession no.
Chromosome 1	3,069,135	63.3	2,851	12	58	AP024322
Chromosome 2	1,492,200	63.7	1,368	3	5	AP024323
Chromosome 3	848,037	63.1	790	3	2	AP024324
pNK81	1,684,880	62.9	1,476	0	0	AP024325
pNK82	1,015,131	60.2	974	0	1	AP024326
pNK83	671,923	63.0	628	0	1	AP024327
pNK84	435,466	59.3	458	0	0	AP024328
Total	9,216,772	62.7	8,545	18	67	

### Data availability.

The complete genome sequence of *Caballeronia* sp. strain NK8 has been deposited in DDBJ/EMBL/GenBank under the accession numbers AP024322 to AP024328, and the raw sequence reads have been deposited under the accession numbers DRA010815 and DRA010852. The BioProject accession number is PRJDB10322.
